# EphA4 Blockers Promote Axonal Regeneration and Functional Recovery Following Spinal Cord Injury in Mice

**DOI:** 10.1371/journal.pone.0024636

**Published:** 2011-09-13

**Authors:** Yona Goldshmit, Mark D. Spanevello, Sophie Tajouri, Li Li, Fiona Rogers, Martin Pearse, Mary Galea, Perry F. Bartlett, Andrew W. Boyd, Ann M. Turnley

**Affiliations:** 1 Centre for Neuroscience, The University of Melbourne, Parkville, Victoria, Australia; 2 Queensland Institute of Medical Research, Herston, Queensland, Australia; 3 Queensland Brain Institute, The University of Queensland, Brisbane, Queensland, Australia; 4 Department of Medicine, The University of Queensland, Brisbane, Queensland, Australia; 5 CSL Limited, Parkville, Victoria, Australia; 6 Physiotherapy Department, The University of Melbourne, Parkville, Victoria, Australia; Charité-Universitätsmedizin Berlin, Germany

## Abstract

Upregulation and activation of developmental axon guidance molecules, such as semaphorins and members of the Eph receptor tyrosine kinase family and their ligands, the ephrins, play a role in the inhibition of axonal regeneration following injury to the central nervous system. Previously we have demonstrated in a knockout model that axonal regeneration following spinal cord injury is promoted in the absence of the axon guidance protein EphA4. Antagonism of EphA4 was therefore proposed as a potential therapy to promote recovery from spinal cord injury. To further assess this potential, two soluble recombinant blockers of EphA4, unclustered ephrin-A5-Fc and EphA4-Fc, were examined for their ability to promote axonal regeneration and to improve functional outcome following spinal cord hemisection in wildtype mice. A 2-week administration of either of these blockers following spinal cord injury was sufficient to promote substantial axonal regeneration and functional recovery by 5 weeks following injury. Both inhibitors produced a moderate reduction in astrocytic gliosis, indicating that much of the effect of the blockers may be due to promotion of axon growth. These studies provide definitive evidence that soluble inhibitors of EphA4 function offer considerable therapeutic potential for the treatment of spinal cord injury and may have broader potential for the treatment of other central nervous system injuries.

## Introduction

In addition to inhibitory molecules associated with myelin and astrocytes, including Nogo, myelin-associated glycoprotein and chondroitin sulfate proteoglycans [1], [2], [3], [4], [5], [6], upregulation of developmental axon guidance molecules, such as semaphorins and members of the Eph receptor tyrosine kinase family, have been shown to play a role in inhibition of axonal regeneration following central nervous system injury [7], [8], [9], [10]. EphA4 expression is upregulated following spinal cord injury [11], [12], [13] and EphA4 null mice show substantially decreased astrocytic gliosis, concomitant with extensive axonal regeneration and recovery of function [12].

Based on the null mouse results, we postulated that blockade of EphA4 function could promote repair following spinal cord injury in wildtype mice. Eph receptors and their ephrin ligands are membrane bound, and activation of the receptor requires clustering within the cell membrane [14]. Artificial Eph receptor activation is achieved by stimulating with soluble ephrin-immunoglobulin Fc fusion proteins that have been clustered together using anti-Fc antibodies [15], [16]. Clustered ephrin-A5-Fc promotes EphA4 phosphorylation and downstream signaling in astrocytes and in neurons, inhibiting neurite outgrowth [12], [17]. Conversely, if the ephrin-Fc or Eph-Fc proteins are unclustered, they antagonize Eph:ephrin interactions [15], [16], [18], resulting in enhanced neurite outgrowth when neurons are grown in the presence of EphA4 [7], [19].

In the current study, we investigated whether inhibition of EphA4 *in vivo* is of therapeutic benefit following spinal cord injury. Two different blockers of EphA4 were examined for their ability to promote axonal regeneration and improve functional outcome following spinal cord hemisection in wildtype mice. These were soluble unclustered ephrin-A5-Fc and soluble unclustered EphA4-Fc. Ephrin-A5-Fc potentially saturates both endogenous EphA4, preventing its activation, and its other high-affinity binding partners, EphA3, EphA5, EphA6 and EphA7 [20]. We have previously shown that ephrin-A5-Fc can block EphA4 activation and hence inhibition of neurite outgrowth [12]. Conversely, soluble EphA4 receptor (EphA4-Fc) can bind to both A- and B-type ephrin ligands [21]. By competitively binding to endogenous ephrin ligands, EphA4-Fc prevents ephrin-induced cell-bound EphA4 activation [22]. Due to the promiscuous nature of EphA4, which interacts with almost all of the ephrin ligands, we hypothesized that EphA4-Fc would be the more effective EphA4 blocking agent *in vivo*. Administration of either ephrin-A5-Fc or EphA4-Fc to wildtype mice for 2 weeks following spinal cord injury resulted in substantial axonal regeneration and functional improvement, indicating that blocking of EphA4 interactions is a viable therapeutic option for the treatment of spinal cord injury.

## Results

### Soluble unclustered ephrin-A5-Fc and EphA4-Fc both block EphA4 activation *in vitro*

**Figure 1:**
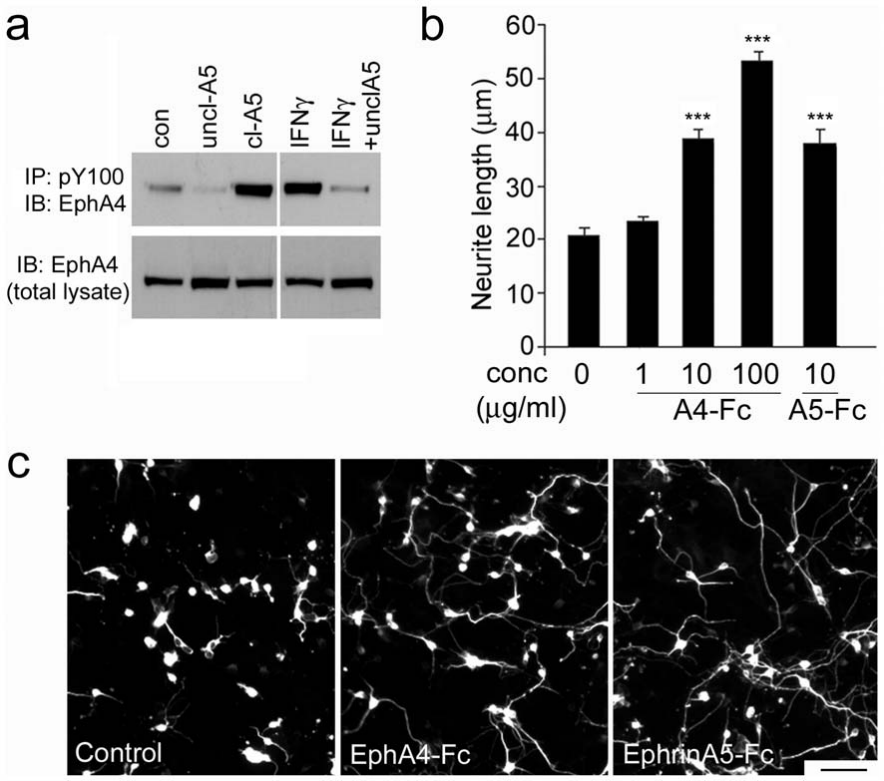
Unclustered ephrin-A5-Fc and EphA4-Fc promote neurite outgrowth. (A) Immunoprecipitation (IP) of phosphotyrosine containing proteins using anti-pY100 antibody followed by immunoblot (IB) for EphA4 showed that unclustered ephrin-A5-Fc (uncl-A5) inhibits basal and IFNγ-induced EphA4 receptor phosphorylation in cultured astrocytes, whereas clustered ephrin-A5-Fc (cl-A5) upregulates EphA4 receptor phosphorylation. (B–C) Inhibition of neurite outgrowth on astrocytes was blocked in a dose-dependent manner by addition of unclustered EphA4-Fc; 10 µg/ml of EphA4-Fc and ephrin-A5-Fc were used in (C). Results in B show mean±SEM, ***p<0.001, using one-way ANOVA with Tukey's multiple comparison test, from *n*≥100 neurons per condition, representative of n = 3 experiments. Scale bar in C, 100 µm.

We have previously shown that astrocytes in culture express EphA4 and that the EphA4 is phosphorylated by addition of clustered ephrin-A5-Fc or a number of inflammatory cytokines, including interferon gamma (IFNγ) [12]. In this study, we therefore first demonstrated that basal EphA4 phosphorylation was blocked by the addition of unclustered ephrin-A5-Fc to the astrocyte cultures, in contrast to clustered ephrin-A5-Fc that has previously been shown to promote EphA4 phosphorylation (Fig. 1a). To test the effectiveness of unclustered ephrin-A5-Fc at inhibiting increased levels of EphA4 phosphorylation we used IFNγ rather than clustered ephrin-A5-Fc, to increase EphA4 phosphorylation. This was to prevent possible unbound anti-human Fc antibodies, present in the clustered ephrin-A5-Fc, from clustering the blocker and making it become an EphA4 activator instead. Addition of unclustered ephrin-A5-Fc inhibited the interferon-induced increase in EphA4 phosphorylation (Fig. 1a). To test the ability of EphA4-Fc to act as a blocker, we cultured embryonic day 16 cortical neurons, which express ephrins, on the EphA4-expressing astrocytes. Similar to what we have previously shown for ephrin-A5-Fc [12], addition of unclustered EphA4-Fc promoted dose-dependent neurite outgrowth (*p*<0.001, F_4,1810_ = 25) (Fig. 1b, c). Therefore, both the soluble EphA4 ligand (ephrin-A5-Fc) and the soluble EphA4 receptor (EphA4-Fc) are able to block EphA4 activation *in vitro*.

### Soluble unclustered ephrin-A5-Fc and EphA4-Fc both diminish astrocytic gliosis

**Figure 2:**
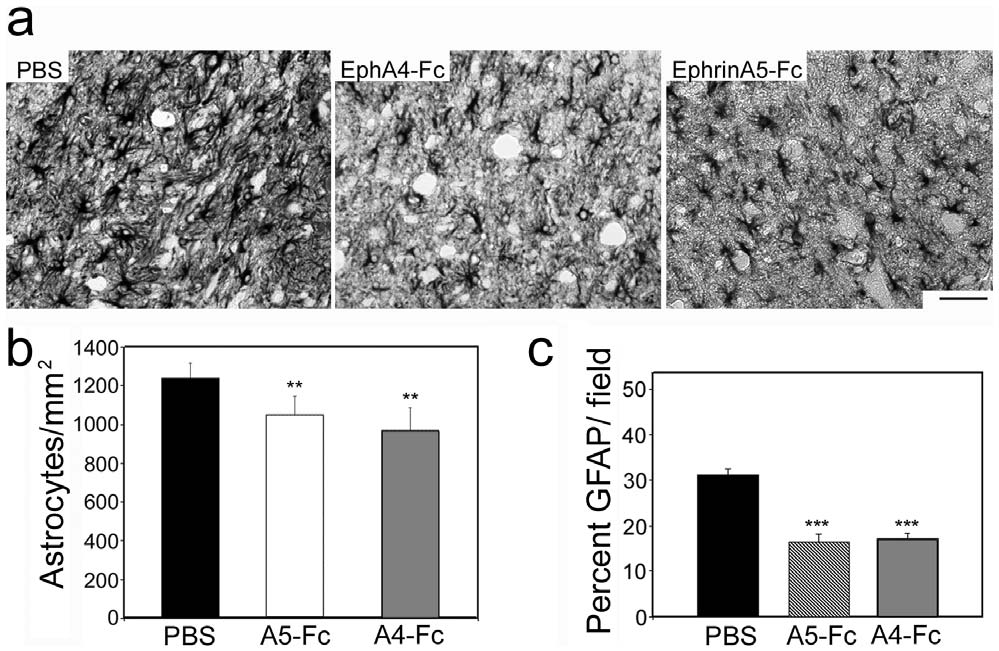
Unclustered ephrin-A5-Fc and EphA4-Fc partially reduce astrocytic gliosis. (A–C) Immunohistochemical analysis of GFAP expression 4 days after spinal cord hemisection and following 3 days administration of Fc fusion protein indicated that, (A) compared to PBS treatment (*n* = 6), ephrin-A5-Fc (*n* = 3) and EphA4-Fc (*n* = 3) treatment decreased astrocytic gliosis. There were significant decreases in the number of astrocytes (B) and GFAP density at the lesion site was reduced following ephrin-A5-Fc or EphA4-Fc treatment (C). Results in B and C show mean±SEM, **p<0.01, ***p<0.001, using one-way ANOVA with Tukey's multiple comparison test. Scale bar in A, 100 µm.

**Figure 3:**
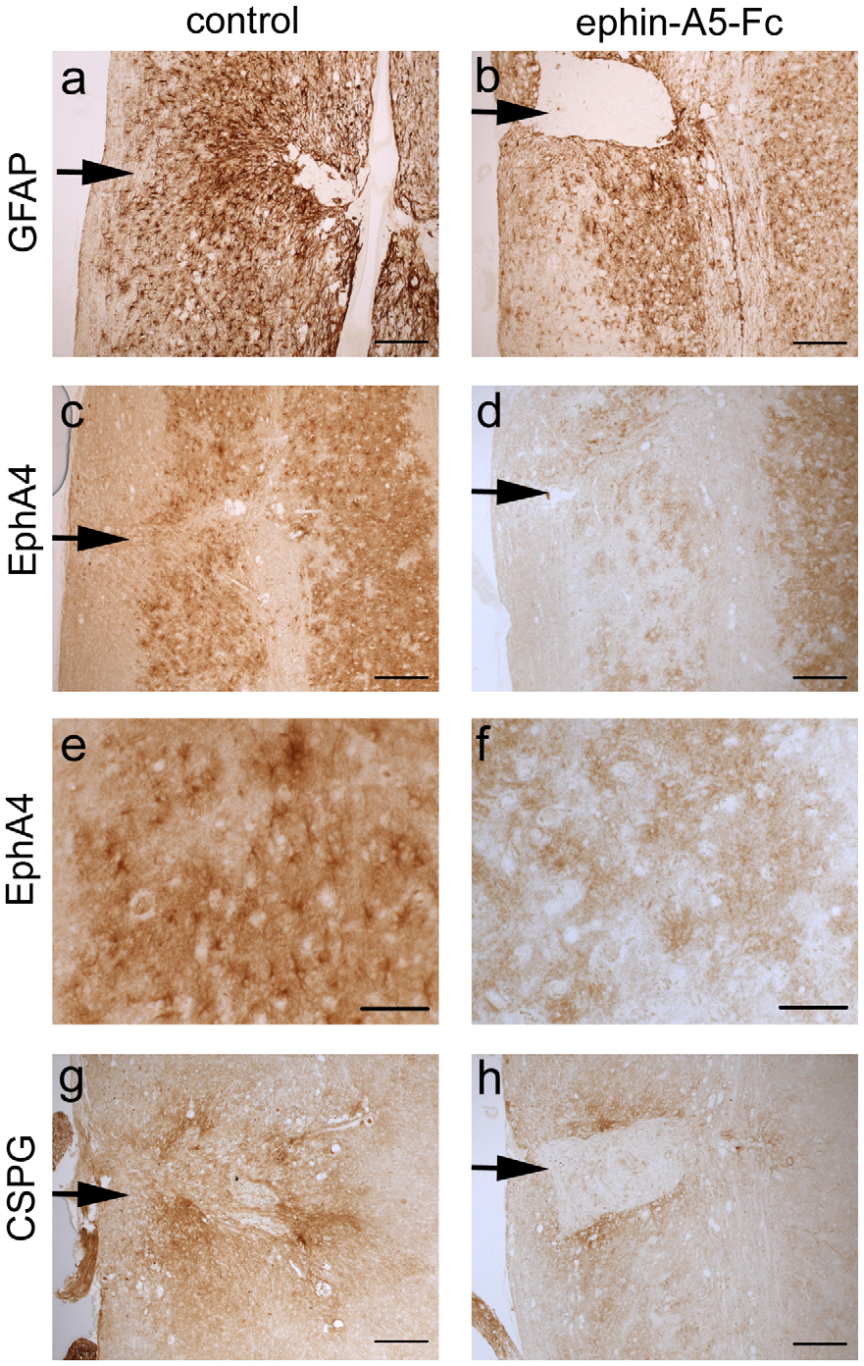
EphA4 immunostaining is decreased adjacent to injury site in ephrin-A5-Fc treated mice. Immunohistochemical analysis of (A,B) GFAP, (C–F) EphA4 and (G,H) CSPG expression at 2 weeks after spinal cord hemisection and 2 weeks of ephrin-A5-Fc treatment indicated that there was robust GFAP staining in both treated and control mice (A,B). However, in treated mice ipsilateral astrocytes adjacent to the injury site had markedly decreased EphA4 staining (C–F). There was also moderately decreased CSPG staining adjacent to the injury site of treated mice (G,H). Arrows indicate lesion site. Scale bars in A–D, G,H, 100 µm; E,F, 50 µm.

As the physical barrier imposed by the development of the gliotic scar is known to impede axonal regeneration following spinal cord injury, we examined the effect of the EphA4 blockers on astrocytic gliosis. We analyzed glial fibrillary acidic protein (GFAP) expression by immunohistochemistry at 4 days after the injury, following a 3-day treatment, to assess early effects on gliosis. Although all animals showed a strong astrocytic response, mice treated with EphA4-Fc or ephrin-A5-Fc showed a significant, but modest, decrease in GFAP positive astrocyte numbers (*p*<0.001, F_2,127_ = 47.27) and these astrocytes had fewer GFAP-positive processes, leading to a decreased level of GFAP expression (*p*<0.001, F_2,9_ = 49.27) compared to controls (Fig. 2a–c). Astrocytic gliosis was still prominent as assessed by GFAP expression at 2 weeks after injury and following 2 weeks of ephrin-A5-Fc treatment (Fig. 3). However, immunostaining for EphA4 showed that, unlike in control mice, there was a marked reduction in EphA4 immunostaining surrounding the injury site, which was not observed further away from the injury site or on the contralateral side (Fig. 3). Immunostaining for the glial scar marker chondroitin sulfate proteoglycan (CSPG) also appeared to be diminished in the treated spinal cords (Fig. 3).

### Soluble unclustered ephrin-A5-Fc and EphA4-Fc both promote regeneration by 6 weeks *in vivo*

**Figure 4:**
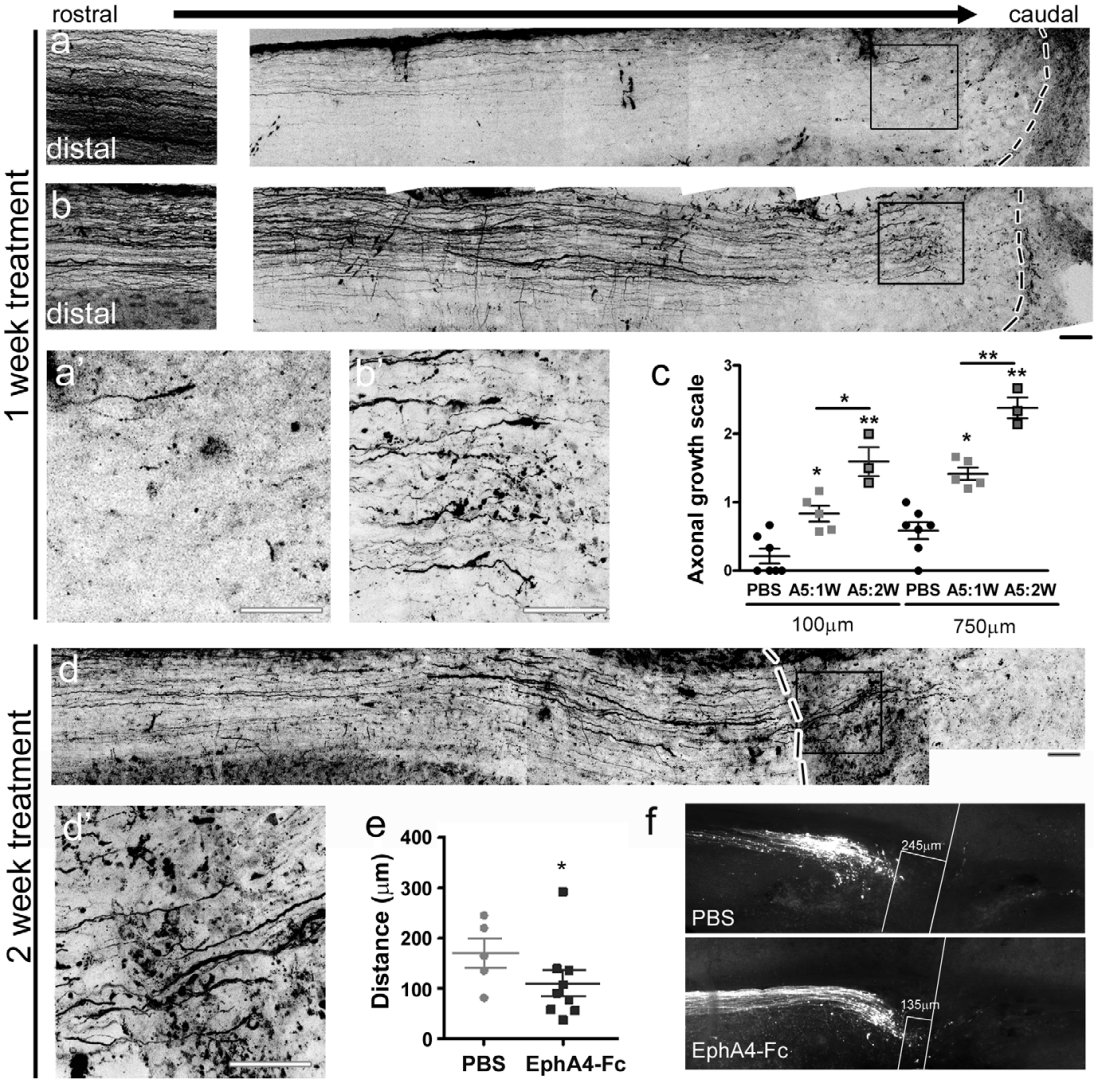
Axonal regrowth towards the lesion site in ephrin-A5-Fc-treated spinal cords 2 weeks after injury. A montage of confocal images 2 weeks after injury from representative sections anterogradely traced with Fluoro-ruby showing that (A) PBS-treated mice had few labeled axons rostral to the lesion site and that (B) one week of ephrin-A5-Fc treatment increased the number of axons proximal to the lesion site. Axons in the ephrin-A5-Fc-treated mice had robust growth cones. Boxes show enlarged regions in a′ and b′. Scale bars for A, B, A′, B′, 50 µm. Dotted lines in A, B indicate the border of the lesion site and the right hand side of panels is caudal to the lesion site (C) Axonal regrowth was determined by semi-quantitative analysis of axons 100 µm or 750 µm rostral to the lesion site after ephrin-A5-Fc (A5) treatment for 1 week (1 W) or 2 weeks (2 W): ordinal scale 0 = no axons; 1 = fewer than 10 axons; 2 = 10–50 axons; 3 = more than 50 axons per section (from n≥5 sections per spinal cord). *p<0.05, **p<0.01, ***p<0.001 compared to the PBS-treated control, using one-way ANOVA with Tukey's multiple comparison test. (D) Two weeks treatment with ephrin-A5-Fc promoted substantial axonal regeneration into the lesion site; box enlarged in D′. Scale bars D, D′, 50 µm. (E) Regrowth of labeled corticospinal tract axons towards the lesion site at 2 weeks, after 2 weeks EphA4-Fc treatment, with distance from center of injury site; *p<0.05. (F) Representative images of labeled corticospinal tract axons 2 weeks after injury, and a 2-week treatment protocol, showing distance to the center of the injury site.

**Figure 5:**
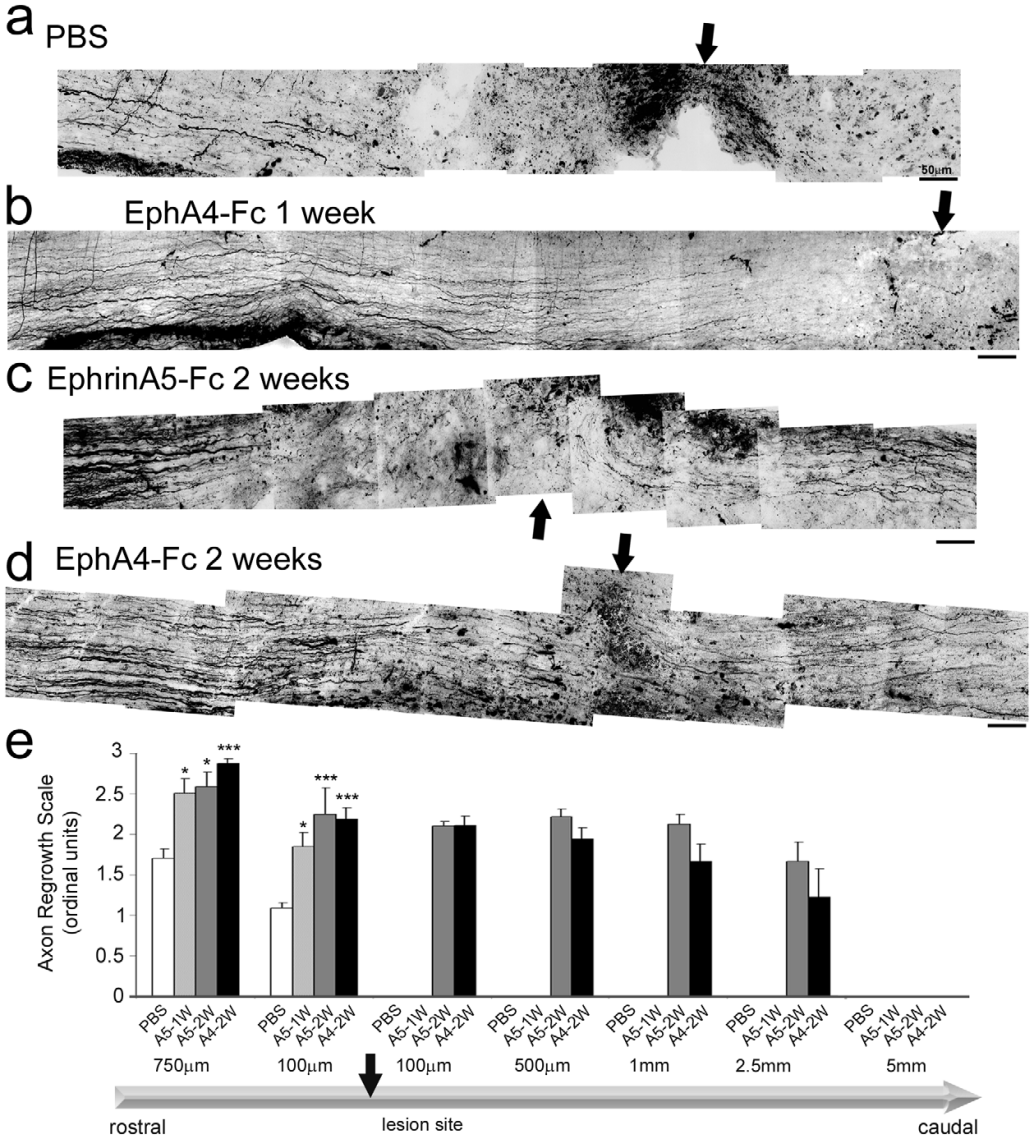
Axonal regeneration in ephrin-A5-Fc- and EphA4-Fc-treated spinal cords 6 weeks after injury. A montage of confocal images, 6 weeks after injury, from representative sections anterogradely traced with Fluoro-ruby showing that (A) PBS-treated mice had few labeled axons rostral to the lesion site. (B) One week of EphA4-Fc treatment increased the number of axons rostral to the lesion site but did not promote regeneration through it. A 2-week treatment with ephrin-A5-Fc (C) or EphA4-Fc (D) was sufficient to promote regeneration through and caudal to the lesion site. Scale bars for A–D, 50 µm. Arrows point to center of lesion site. (E) Axonal regrowth was determined by semi-quantitative analysis of labeled axons rostral and caudal to the lesion site after ephrin-A5-Fc (A5) or EphA4-Fc (A4) treatment for 1 week (1 W) or 2 weeks (2 W): ordinal scale 0 = no axons; 1 = fewer than 10 axons; 2 = 10–50 axons; 3 = more than 50 axons per section (from n≥5 sections per spinal cord). *p<0.05, ***p<0.001 compared to PBS control, using one-way ANOVA with Tukey's multiple comparison test.

Having demonstrated the activity of the EphA4 blockers on the gliotic response, we next investigated their effect on axonal regeneration in the wildtype mice. Anterograde tracing was used to compare the effectiveness of a 1-week versus a 2-week treatment on axonal regeneration at 2 and 6 weeks after the injury. No axons were observed distal to the lesion site 2 weeks after injury in any condition (Fig. 4). Animals that were treated for 1 week with ephrin-A5-Fc displayed increased numbers of axons approaching the lesion site at 2 weeks, compared to controls (Fig. 4a, b, c). The axons in the treated mice also had tip morphology suggestive of robust growth cones, indicating that inhibition of Eph/ephrin signaling reduced growth cone collapse (Fig. 4b′). No further improvement was observed at 6 weeks after injury, in control (Fig. 5a, Fig. S1a) or ephrin-A5-Fc treated mice (Fig. 5b). No axons were observed entering or crossing the lesion site at 6 weeks after injury, although more axons reached the proximal lesion edge of the injury in treated animals compared to controls (Fig. 5e; *p*<0.001, F_3,21_ = 12.61 at 100 µm proximal to the lesion site and *p*<0.001, F_3,21_ = 15.97 at 750 µm).

As treatment for 1 week was not effective at promoting axonal regeneration, we extended the delivery period to 2 weeks to determine whether longer administration of ephrin-A5-Fc or EphA4-Fc would be more effective. We initially examined axonal regeneration immediately after the 2-week delivery period and observed that treatment with ephrin-A5-Fc resulted in increased numbers of axons proximal to the lesion site (Fig. 4c, d; *p*<0.001, F_2,12_ = 24.51 for 100 µm proximal to the lesion and *p*<0.001, F_2,12_ = 44.05 for 750 µm). In addition, a small proportion of axons entered but did not cross the injury site (Fig. 4d′). A similar result was found following a 2-week treatment with EphA4-Fc. Anterograde tracing revealed that most mice in the treated group had axons bordering or within the lesion site (Fig. 4e, f).

Importantly, at 6 weeks after the injury, we observed that administration of ephrin-A5-Fc or EphA4-Fc over 2 weeks resulted in substantial axonal regeneration, with many axons entering and crossing the lesion site to extend distally in both treatment groups (*p*<0.001, F_15,81_ = 25.12; Fig. 5c–e, Fig. S1b,c). To determine if greater regeneration resulted from local administration compared to systemic administration, EphA4-Fc was applied in gelfoam above the lesion site. This resulted in slightly better axonal regeneration than the 1-week intraperitoneal (i.p.) treatment, with many axons entering the lesion site (Fig. S1d). However, as this regimen was not as effective as the 2 week i.p. treatment, it was not pursued further in the current study.

The presence of axons adjacent to the injury site at 2 weeks in treated mice could be due to a lack of axonal die-back or regeneration of axons after die-back. To determine which of these was occurring, cortical axons were anterogradely labeled with Fluoro-ruby 1 week prior to injury. After spinal cord hemisection, EphA4-Fc was administered for 3 days and tissue was taken for analysis at 4 days, a time at which axonal die-back should occur. Compared to the number of labeled axons 1.5 mm from the injury site, at 1 mm control mice (n = 3) had 93±5.5% and EphA4-Fc-treated mice (n = 5) had 97±6.6% the number of axons, whereas at 0.5 mm, there was a non-significant trend towards increased numbers of labeled axons in treated animals (74±13.1% in the EphA4-Fc-treated group (*n* = 5), compared to 54±6.1% in controls (*n* = 3) *p* = 0.22, t-test) (Fig. S2). This suggests that EphA4-Fc treatment did not prevent axonal die-back but rather promoted axon regrowth.

### Soluble unclustered ephrin-A5-Fc and EphA4-Fc both promote functional recovery

**Figure 6:**
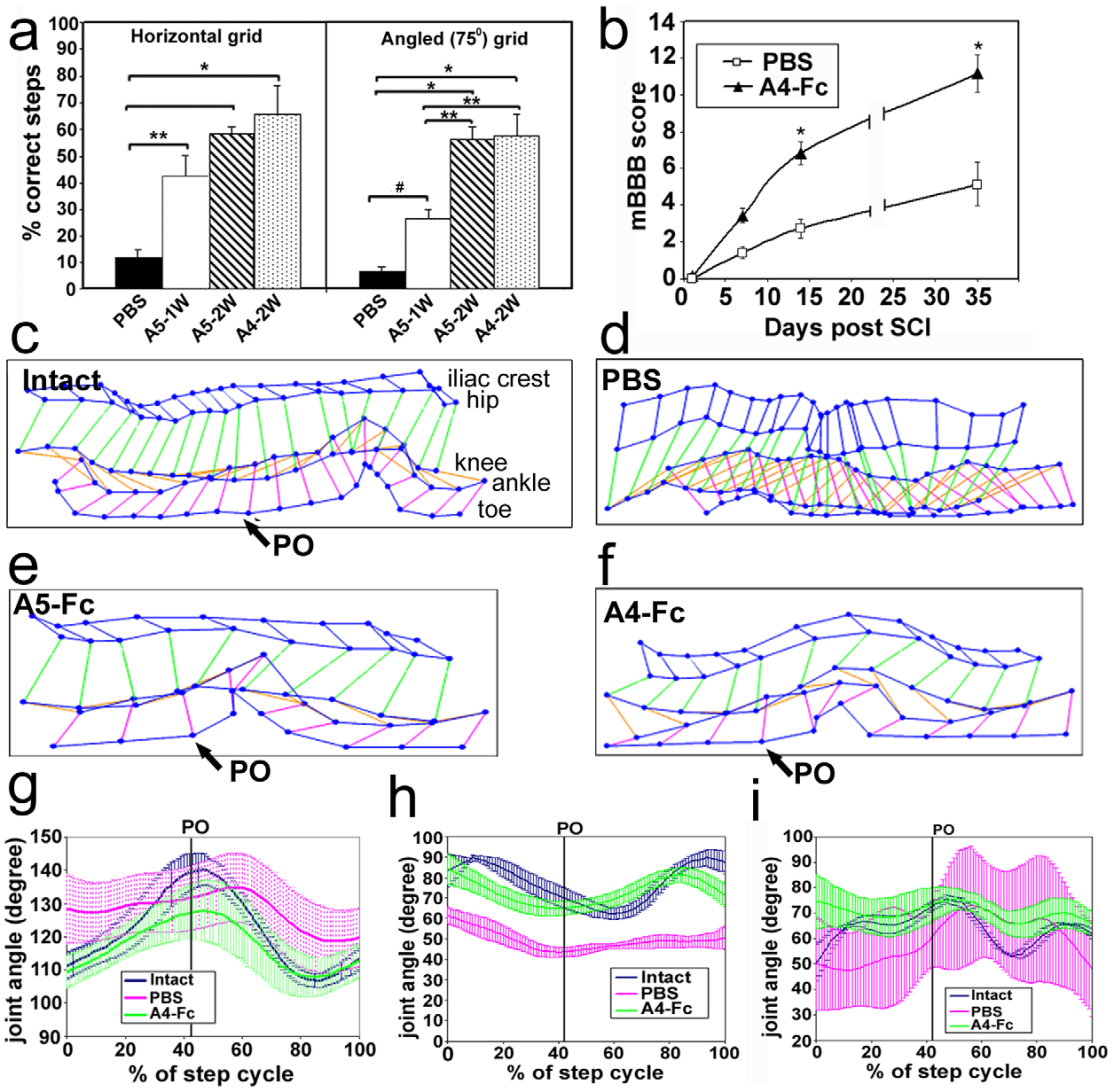
Ephrin-A5-Fc- or EphA4-Fc-treated mice show significant functional recovery 5 weeks after spinal cord injury. (A) Walking and climbing on a grid were significantly improved following 2 weeks treatment with ephrin-A5-Fc (A5-2W) or EphA4-Fc (A4-2W). One week of ephrin-A5-Fc (A5-1W) treatment resulted in significant improvement in grid walking but not in climbing as assessed by ANOVA, although it was significant by t-test (mean±SEM, *p<0.05, ***p<0.001 using one-way ANOVA with Tukey's comparison analysis; #*p*<0.05 using t-test). (B) The mBBB score was measured up to 5 weeks after spinal cord injury (SCI) in PBS- (*n* = 7) and EphA4-Fc- (A4-Fc) (*n* = 6) treated mice. Results show mean±SEM (***p<0.001 comparing treatment groups at the indicated time point using one-way ANOVA with Tukey's multiple comparison test). Kinematics of the left hind limb were analyzed from videotapes of locomotion of mice on the treadmill with reflective markers on the iliac crest, hip joint, knee joint and ankle joints. (C–F) Stick figures of the angles between each joint were used to depict one complete step cycle, with the beginning of the swing phase marked with an arrow for the push off point (PO) of the left hind limb and the pattern obtained for a normal intact mouse at a treadmill speed of 12 m/min (C). Panels (D–F) show a representative pattern for one mouse from each of the PBS (*n* = 7), ephrin-A5-Fc (A5-Fc) (*n* = 2) and EphA4-Fc (A4-Fc) (*n* = 6) groups of mice 5 weeks after spinal cord injury, at a treadmill speed of 12 m/min. Treatment over 2 weeks with ephrin-A5-Fc and EphA4-Fc resulted in approximation of the movement pattern seen in intact animals, including a phase of lifting the paw off the surface. (G–I) Average kinematic profile combined from profiles of EphA4-Fc-treated mice (*n* = 6), of joint angle changes over one complete step cycle of the left hip (G), knee (H) and ankle joints (I) 5 weeks after spinal cord injury in PBS- and EphA4-Fc-treated mice compared to the intact mouse pattern. The point of push off (PO) and the beginning of the swing phase is indicated by a black vertical line through the graph. Results are expressed as mean±SD.

Mice were assessed for functional recovery, as determined by the use of their left hind limb, at 5 weeks, immediately prior to the injection of anterograde tracer. Both ephrin-A5-Fc and EphA4-Fc treatments resulted in a significant improvement in the ability to walk (*p*<0.001, F_3,18_ = 20.04) or climb (*p*<0.001, F_3,18_ = 35.87) on a grid, with animals making far fewer foot falls and showing weight support with the affected left hind limb (Fig. 6a; Videos S1 and S2). Mice treated with EphA4-Fc also showed significant improvement of function based on the mouse modified open-field behavior test (mBBB scale; [23], (Fig. 6b; repeated measures ANOVA, *p*<0.001, F_3,15_ = 92.27) and grasp ability (PBS-treated mice scored 1.1±0.8, indicating partial movement of the paw, with no movement of the toes; EphA4-Fc-treated mice scored 2.2±0.4, indicating partial grasp and slight movement of toes and paw).

Kinematic gait analysis indicated that the treated mice had a more normal looking step cycle pattern, with an obvious swing phase of the paretic hind limb (Fig. 6c–f, Videos S3 and S4). Individual joint movement was analyzed by measurement of joint angle throughout one step cycle in the EphA4-Fc-treated mice compared to untreated mice and intact controls. In treated mice, the hip joint started to approximate normal movement, although the range was diminished (Fig. 6g). The knee joint showed some movement but the timing was altered, with a shorter stance phase (Fig. 6h), whereas untreated mice showed almost no knee movement. Movement of the ankle joint of treated mice was small. However, in untreated mice the effect at the ankle was very variable, ranging from fully flexed (spastic) to dragging (Fig. 6i).

### Prolonged therapeutic delivery and extended recovery following EphA4-Fc treatment

**Figure 7:**
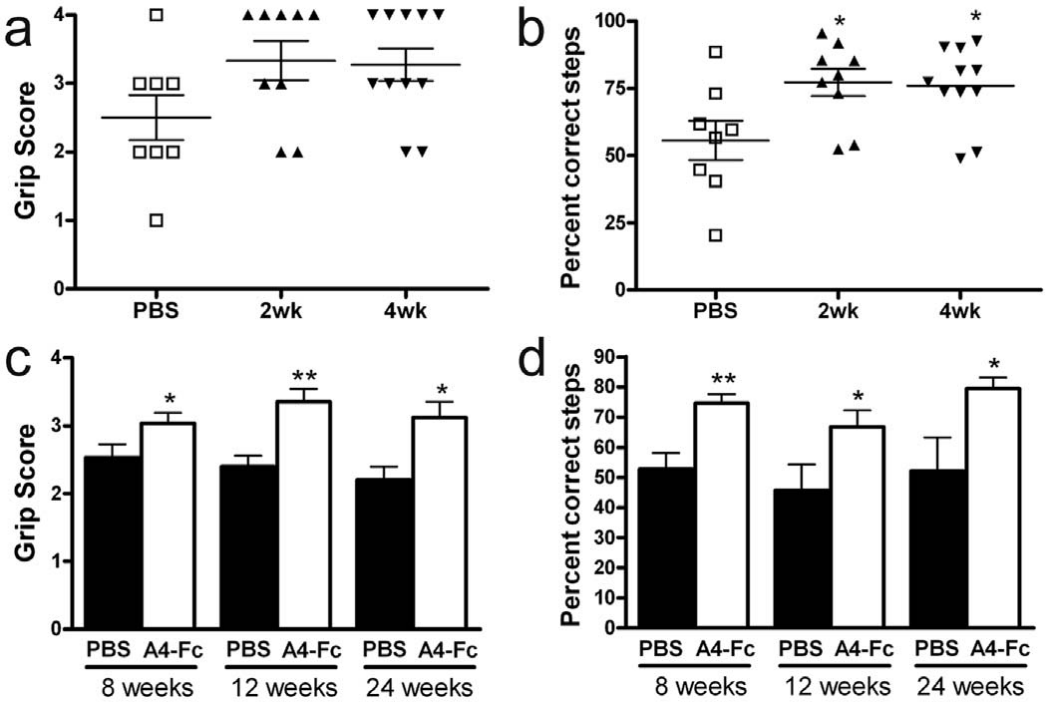
Prolonged treatment with EphA4-Fc and extended recovery. (A, B) To determine whether extending treatment length to 4 weeks promoted greater functional improvement EphA4-Fc was administered for 2 weeks or 4 weeks, after which (A) grasp score and (B) grid climbing accuracy were assessed at 6 weeks post-injury. Both the 2-week and 4-week treatments improved functional outcomes compared to controls, but were not significantly different to each other. (C, D). To determine whether extending the recovery period for longer than 6 weeks would allow further functional improvement, animals were treated with EphA4-Fc for 2 weeks after injury and assessed at 8, 12 and 24 weeks. Analysis of (C) grasp score and (D) grid climbing accuracy showed that no further improvements were observed after 8 weeks in control or treated mice. (mean±SEM, *p<0.05, **p<0.01).

As EphA4-Fc was at least as effective as ephrin-A5-Fc at promoting recovery from spinal cord injury, but with half the number of doses, it was chosen for further analysis. To determine whether more prolonged administration would further enhance recovery, EphA4-Fc was administered for either 2 or 4 weeks and functional recovery was then assessed at 6 weeks. Both grasp score and grid climbing accuracy were improved by EphA4-Fc treatment compared to controls, but there was no significant difference between animals receiving treatment over 2 or 4 weeks. (Fig. 7a,b). To determine whether recovery for longer than 6 weeks would result in further functional improvement, EphA4-Fc was administered for 2 weeks and grasp score and grid climbing accuracy assessed at 8, 12 and 24 weeks post injury. Both grasp score and grid climbing accuracy reached a plateau between 8 and 12 weeks following injury (Fig. 7c, d). These data suggests that a 2-week treatment paradigm is sufficient to maximize functional improvement and that functional improvement observed by 8 weeks is maximal.

## Discussion

The results of the present study have revealed that, following spinal cord hemisection in wildtype mice, a 1-week treatment with an EphA4 blocker was largely ineffective, enhancing axonal regrowth to, but not beyond, the injury site. However, 2 weeks administration of either ephrin-A5-Fc or EphA4-Fc promoted extensive axonal regeneration by 6 weeks, as well as significant behavioral improvement. Although the observed level of improvement was similar with both treatments, the same outcome was achieved using fewer treatments and a lower dose of EphA4-Fc than for ephrin-A5-Fc. This may reflect the promiscuous affinities of EphA4, which is the only A class receptor that binds with high affinity to multiple A and B type ephrins [24], [25]. This then allows EphA4-Fc to block multiple Eph:ephrin interactions [21].

The 1-week treatment protocol was sufficient to increase the number of axons regrowing to the lesion site. This is likely to lead to enhanced function at least to the hip joint, an outcome that was reflected in improvement on the relatively simple horizontal grid test. However, it was not enough to produce significant improvement in the more difficult climbing task, which also requires the ability to lift the leg more precisely, as well as to bear weight. Using the same mouse model of left spinal hemisection, a similar improvement in function, with concomitant regrowth to, but not into or beyond the injury site, was found in response to forced treadmill exercise [26].

Interestingly, a 4-week treatment of EphA4-Fc was no more effective than a 2-week treatment. This may be due to permeability of the blood-brain barrier. Eph:ephrin signaling regulates angiogenesis [27] and blood vessel structure [28], [29], including in the nervous system [30]. We have previously demonstrated that, unlike wildtype mice in which the blood-brain barrier has substantially repaired by 1 week, the blood-brain barrier at the injury site in EphA4 null animals still displays extensive leakage at 2 weeks, with substantial repair only becoming apparent by 3 weeks [30]. Treatment with Eph blockers may thus have extended blood-brain barrier permeability in the wildtype mice, to the point where it allowed the blockers to enter the injury site for 2 weeks, but precluded subsequent access due to barrier repair. This may be due to direct effects on the endothelial cells, as well as on astrocytes that contribute to blood-brain barrier formation [31].

A further, as yet unexplored mechanism which may contribute to the regeneration and recovery promoted by blocking of EphA4 is an effect on inflammation. EphA4 null mice show thymic alterations and T cell defects [32]. Given that blocking of inflammation has also been demonstrated to promote recovery from spinal cord injury [33], [34], it is possible that blocking of EphA4 altered the inflammatory response following injury.

It is interesting to note that the regrowing axons in the treated mice were generally long and relatively straight in appearance, although there was also substantial collateral sprouting and some deviation around the most damaged parts of the injury site. Axons regenerating in response to other factors generally do not follow such a straight line [35]. As EphA4 is upregulated on astrocytes following injury, we hypothesize that its expression may modulate axon:astrocyte interactions.

The mechanism by which the Eph-blocking treatments promoted regeneration appears to be primarily at the level of the axonal growth cone, blocking EphA4:ephrin interactions that would normally result in growth cone collapse. Given the bidirectional signaling that occurs for Eph:ephrin interactions [36], an ephrin expressed on the axonal growth cone may induce growth cone collapse in response to EphA4 expression on the astrocytes. In addition, EphA4 expressed on the growth cone may also induce growth cone collapse in response to ephrins expressed on astrocytes [37] or myelin [38].

We have previously reported a strong decrease in astrocytic activation and proliferation in EphA4 null mice (generated by homologous recombination) following a lateral hemisection injury [12]. However, similar to previous reports using other EphA4 antagonists after spinal cord injury, we show here that astrocytic gliosis, as defined by GFAP expression, is not robustly affected [39], [40]. This is similar to a recent examination of a dorsal hemisection injury in EphA4 null mice generated using gene trap methodology, which did not show an effect on gliosis [41]. Recent *in vitro* experiments showed that effects of EphA4 on GFAP expression in cultured astrocytes were relatively modest, while effects on the astrocyte actin cytoskeleton and focal adhesion were more pronounced [42]. Therefore, while the effect of EphA4 on GFAP expression requires further elucidation, it is clear that regulation of EphA4 activity modulates broader astrocyte reactivity. In the current study, the difference in level of GFAP expression was modest but significant and may reflect the substantial biological activity of EphA4-Fc delivered repeatedly compared to small peptide or antisense approaches. In particular, our results suggest that, even in the presence of gliosis as defined by upregulation of GFAP expression, functional recovery and axonal regeneration can still occur. Of note, immunostaining for EphA4 was decreased adjacent to the injury site in treated spinal cords, which indicates that a major repulsive signal on the surface of the reactive astrocytes was diminished in the treated animals close to the site of injury and may account for the ability of axons to cross and extend past the injury site.

Functional blocking using soluble ligands or receptors appears to be required as partial downregulation of EphA4 expression using antisense oligonucleotides has proven ineffective at promoting regeneration following spinal cord injury [43]. Furthermore, use of an EphA4 peptide antagonist [44] following spinal cord injury was only partially effective [39]. The ability of the two different Fc fusion proteins used in the present study to physically block interactions between multiple Eph receptors and ephrin ligands may also be an important part of their mechanism of action and allow for wider inhibition than the more constrained peptides.

One of the mechanisms downstream of Eph receptor activation leading to growth cone collapse is likely to involve the Rho/Rac/Cdc42 cellular apparatus, with Rho-GTPase inhibition previously shown to promote recovery from spinal cord injury [45], [46], [47]. Rho is activated downstream of repulsive guidance molecules and inhibits axonal regeneration through the stimulation of growth cone collapse [46], [48], [49]. The equilibrium between Rho, Rac and Cdc42 is shifted towards Rho activation following ephrin activation of EphA4 and causes growth cone collapse [50]. Blocking Rho alters the bias towards Rac and Cdc42 activity, promoting axonal outgrowth [50], [51], [52]. However, the reported functional recovery after injury following direct Rho inhibition by use of Rho pathway inhibitors occurs within 1–2 days after injury [45], which is earlier than the recovery we observe with EphA4 antagonism. This suggests that directly inhibiting Rho may affect other cellular processes, such as decreasing apoptosis [46]. Future experiments will assess whether Rho activation is diminished in spinal cords after treatment with the Fc fusion protein blockers.

Expression of a number of other developmental repulsive axon guidance molecules has also been shown to be upregulated following spinal cord injury, with semaphorins in particular appearing to have an effect similar to that observed for EphA4 [8], [9], [53], [54]. Blocking of Wnt-5a-Ryk signaling also promotes axonal regrowth in the corticospinal tract and enhances functional recovery [55]. Blocking of EphA4 is highly effective at promoting regeneration of multiple axonal tracts, most likely due to its high promiscuity in binding to all ephrins and the widespread expression of EphA4 and different ephrin ligands on a wide range of neurons, as well as astrocytes, myelin, meningeal and endothelial cells [7]. However, a combined therapy that includes blocking of semaphorins, for example, may be even more effective at promoting axonal regeneration and further functional recovery.

In summary, the effectiveness of both ephrin-A5-Fc and EphA4-Fc treatments provides the first definitive evidence that soluble inhibitors of EphA4 function can be used therapeutically to promote recovery from spinal cord injury. Not only do these inhibitors hold promise for treatment of spinal cord injury, but also they are likely to promote recovery following brain trauma or stroke.

## Materials and Methods

### Protein expression and purification

To produce the Fc fusion proteins, the extracellular domains of mouse EphA4 (amino acids 1–546 of NP_031962.2) and human ephrin-A5 (amino acids 1–201 of NP_001953.1) were cloned into the pTIgBOS vector [56]. These constructs were used to stably transfect the Chinese hamster ovary (CHO) cell line (ATCC, USA) to produce EphA4-Fc and ephrin-A5-Fc respectively. High producer lines were selected and routinely grown in GIBCO Hybridoma serum-free medium (Invitrogen). The fusion protein was purified from cell culture supernatants by Protein A Sepharose (Amersham Biosciences) affinity chromatography. Purity was assessed by SDS-PAGE and determined to be >95% for EphA4-Fc and >75% for ephrin-A5-Fc.

### Astrocyte and neuronal cultures and neurite length measurement

For analysis of EphA4 phosphorylation, clustered and unclustered ephrin-A5-Fc (1.5 µg/ml and 10 µg/ml respectively) was added to monolayers of primary astrocytes (cultured from postnatal day 1–3 cortices) for 1 hour. To induce EphA4 phosphorylation in the absence of added clustered ephrin-A5-Fc, IFNγ (100 U/ml, BD Biosciences, Sydney, Australia) was then added to the astrocytes for 1 hour. Phosphoproteins were immunoprecipitated and EphA4 levels quantified as previously described [12]. For analysis of neurite length, embryonic day 16 cortical neurons were plated at 5,000 cells/well in chamber slides (Falcon; BD Biosciences) on confluent monolayers of astrocytes pretreated for 1 hour with unclustered ephrin-A5-Fc (10 µg/ml) or unclustered EphA4-Fc (0 µg/ml, 1 µg/ml, 10 µg/ml or 100 µg/ml). After 2 days, cells were fixed and immunostained for the neuronal marker βIII-tubulin (1∶2000, Promega). Images of neurites were digitally captured using a Zeiss Axioplan 2 imaging fluorescence microscope, fitted with a Zeiss Axiocam HRc digital camera and Zeiss Axiovision Release 4.5 software. Neurite length was measured by HCA-Vision software (Neurite Analysis Module V1.0.1, CSIRO Mathematical and Information Sciences, Sydney, Australia). The data were analyzed by one-way ANOVA followed by Tukey's post-comparison analysis, and presented as mean±SEM.

### Ethics statement

All procedures were approved by the University of Melbourne (approval #0706768) or the University of Queensland (approval #SBMS/466/08) Animal Ethics Committee in accordance with the Australian Code of Practice for the Care and Use of Animals for Scientific Purposes.

### Animals and surgical procedures

Spinal cord injury surgeries were performed on adult (8–12 week old) male C57BL/6 mice. Mice were anesthetized with a mixture of ketamine and xylazine (100 mg/kg and 10 mg/kg, respectively), after which they received a complete left hemisection at vertebral level T12 as previously described [12]. Briefly, after laminectomy, a left hemisection was made at T12 with a fine ophthalmic blade, after which the overlying muscle and skin were sutured. The mice were randomly assigned to the control (phosphate-buffered saline; PBS), unclustered ephrin-A5-Fc or unclustered EphA4-Fc groups prior to recovery from the anesthetic and were analyzed at various time points up to 24 weeks post-injury. Administration of the PBS, ephrin-A5-Fc or EphA4-Fc by i.p. injection began 2 hours following surgery. To ensure competitive saturation of endogenous Eph receptor or ephrin ligands, high doses of EphA4-Fc and ephrin-A5-Fc were used. Ephrin-A5-Fc has a relatively quick clearance rate [57] and in preliminary experiments was injected daily for 3 days, 1 week or 2 weeks at quantities that would surpass 250 nM (680 µg/dose) at dosing. Analysis of EphA4-Fc clearance showed sustained levels with slower clearance and serum concentrations sufficient for saturation (>50 nM) after 48 hours with doses of 1 mg (Fig. S1). To account for this, EphA4-Fc was injected as a larger initial dose (1 mg) at 2 hours, followed by a lower dose (500 µg) every second day for 3 days, 2 weeks or 4 weeks. Administration of these Fc fusion proteins was well tolerated by the mice. All treated animals appeared healthy and active and unaffected by the amount of protein injected.

Tetramethylrhodamine- or fluoroscein-conjugated dextrans (Fluoro-ruby and Fluoro-emerald, respectively; Invitrogen) were used as anterograde tracers to examine the extent of regeneration of lesioned lateral white matter tract axons or corticospinal tract axons. Tracing was performed after behavioral analyses and 1 week prior to tissue collection. For lateral white matter tract axons, the mice were anesthetized and the spinal cord was exposed in the cervical region as described above. The tracer was injected into the spinal cord at the level of the cervical enlargement, ipsilateral to the lesion (three injection points, 0.3 µl at 50 mg/ml each) and left for another 7 days as described previously [12]. For the axon die-back analysis, lateral white matter tract tracing was performed 1 week prior to spinal cord hemisection, as described above. Mice were allowed to recover for 7 days before undergoing a lateral hemisection injury and treatment with EphA4-Fc as described. Mice were sacrificed 4 days after spinal cord injury for analysis.

### Immunohistochemistry and astrocyte counts

Standard immunohistochemical procedures, using rabbit anti-glial fibrillary acidic protein (GFAP; 1∶500, Dako), mouse anti-CSPG (1∶200; Sigma) or rabbit anti-EphA4 (F88 antiserum, prepared in Bartlett laboratory against a peptide corresponding to amino acids 938–953 of the intracellular SAM domain of EphA4 (GenBank accession number NM007936)) were performed on longitudinal sections of spinal cord at either 4 days or 2 weeks after injury, following a 3-day or 2-week treatment respectively. The total number of GFAP-expressing astrocytes at 4 days was counted in a 0.25 mm^2^ grid at the lesion site, in every third serial longitudinal 10 µm section. Only astrocytes in which the nucleus was present in the section were included in the cell density counts. GFAP density was measured at ×1000 magnification using Image J software (Wayne Rasband, National Institutes of Health). The GFAP density was averaged from at least five boxes per section and five sections per spinal cord, with the results being presented as the percentage of each field showing GFAP expression.

### Analyses of anterograde tracing

Longitudinal serial sections for analysis of anterograde tracing were cut on a freezing microtome at 75 µm and then examined using fluorescence and confocal microscopy. The number of axons reaching the lesion site was counted in the area at the edge of the lesion site (100 µm) and 750 µm proximal to the lesion site under ×400 magnification, from at least five sections per spinal cord. Completeness of the hemisections was confirmed by examination of hematoxylin and eosin (H&E) or GFAP-labeled sections. To determine the extent of regrowth past the lesion site, the percentage of mice in which axons crossed the lesion site and were present 100 µm, 1 mm, 2.5 mm or 5 mm distal to the lesion site was determined. Comparison of the average number of labeled axons per section between the groups was performed using one-way ANOVA. Images of traced lateral white matter tract axons were obtained from Z-stacks generated by confocal microscopy, using a Bio-Rad MRC1024 confocal scanning laser system installed on a Zeiss Axioplan 2 microscope and Confocal Assistant version 4.02 software. For the axon die-back analysis, the total number of axons observed at 0.5 mm and 1.0 mm were expressed as a percentage of the number of axons observed at 1.5 mm proximal to the injury site. Counts were cumulative from the five sections with the highest counts. Images of labeled axons were taken with a Zeiss Axio Imager and AxioVision v4.8 software. Photomontage figures were produced using Adobe Photoshop 6.0.

### Behavioral analyses

The open-field locomotion score was evaluated using the modified Basso-Beattie-Bresnahan (mBBB) scoring system of 20 points [23]. Ability to walk/climb on a horizontal and angled grid and grasp strength were performed as previously described [12]. The data were presented as mean±SEM and intergroup comparisons analyzed by one-way ANOVA followed by Tukey's post comparison analysis. In the case of the mBBB analysis, repeated measures ANOVA was used to examine improvement over time within groups.

### Kinematic gait analysis

Locomotion of the left hind limb was recorded prior to the lesion and at 5 weeks after left hemisection, essentially as we previously described [26]. The left hind limb was shaved and reflective markers (3 mm hemispherical; B&L Engineering) were placed over the iliac crest, the femoral head (hip joint), the knee joint and the ankle joint. The lateral metatarsophalangeal joint (little toe) was used as the 5^th^ point. A video camera (Sony HDR-HC1) operating at 25 frames per second (shutter rate = 1/1250 sec) captured the sagittal plane motion of the mice (left hand side) while they walked on the treadmill. The camera was located 1 m from the center of the walkway (center of lens level with treadmill) with a field of view of approximately 32 cm (length)×24 cm (height). The camera lens was leveled in three planes (sagittal, frontal and transverse) to minimize perspective and parallax error.

The treadmill speed was set at 12 m/min. The location of the reflective markers and the 5^th^ metatarsophalangeal joint were digitized directly from the video image using the Peak Motus Motion Measurement System (Peak Performance Technologies). This system captures 50 images or fields for each second (sample rate = 50 Hz). The raw spatial coordinate data were filtered (smoothed) using a 4^th^ order Butterworth digital filter with a cut-off frequency of 5 Hz. The kinematics of several full steps was then analyzed for each mouse. Kinematic data were time-normalized and ensemble averaged across the animals for each group. Inter-subject average kinematic profiles were generated by calculating the mean±SEM of the joint angles at 4% intervals over the stride period.

### Statistics

All statistics were performed using GraphPad Prism 4.0 software. Analysis of treatment effects was performed using one-way ANOVA followed by Tukey's post-hoc test for group comparisons or unpaired t-tests for individual comparisons, with significance set at *p*<0.05.

## Supporting Information

Figure S1 **Additional examples of anterograde tracing 6 weeks after spinal cord injury.** Spinal cords from (**a**) PBS control, (**b**) ephrin-A5-Fc treated and (**c**) EphA4-Fc treated mice. Arrows indicate lesion site. (**d**) Local administration of EphA4-Fc does not promote axonal regeneration through the lesion. Immediately following spinal cord injury, 100 µg EphA4-Fc or human IgG was added in a 10 µl solution to 3×4 mm gelfoam and placed directly above the injury site. Local administration of EphA4-Fc by saturated gelfoam resulted in slightly better axonal regrowth than the 1-week i.p. treatment, with many axons entering the lesion site (white arrows), but it was not as effective as the 2-week i.p. treatment. Scale bars in a-c, 50 µm; d, 200 µm. (**e**) Analysis of EphA4-Fc clearance. EphA4-Fc (1 mg) was injected i.p. and blood samples from three animals were taken at 24 hours, 48 hours and 7 days. One uninjected animal served as a baseline control. Serum was prepared from the blood samples and circulating EphA4-Fc was detected by ELISA. Briefly, purified anti-mouse EphA4 (IF9) monoclonal antibody was bound to EIA plates. Reference EphA4-Fc was diluted from 200 ng/ml to 0 ng/ml and the serum was diluted 1∶1000 and 1∶2000. All standards and samples were added in triplicate. Following washing, bound EphA4-Fc was detected with anti-human IgG-HRP and SIGMAFAST™ OPD colorimetric substrate. A 4^th^-order polynomial standard curve was generated (r^2^>0.999) and used to calculate the serum levels of EphA4-Fc. Data are presented as mean±SEM.
(TIF)

Figure S2 **Anterograde tracing of spinal cords that were labeled prior to spinal cord hemisection.** Anterograde tracing of spinal cords at 4 days post-injury, labeled 1 week prior to injury shows that there was axonal die-back in control and treated mice. Arrow indicates injury site. Scale bar, 500 µm.
(TIF)

Video S1 **Control mice on climbing grid 5 weeks after spinal cord injury.** Mice were assessed for functional recovery, as determined by the use of their left hind limb, at 5 weeks after spinal cord hemisection. Control mice were unable to effectively use their left hindlimb to climb an angled grid.
(WMV)

Video S2 **Ephrin-A5-Fc-treated mice 5 on climbing grid weeks after spinal cord injury.** Mice were assessed for functional recovery, as determined by the use of their left hind limb, at 5 weeks after spinal cord hemisection. Ephrin-A5-Fc treated mice were able to use their left hindlimb to climb an angled grid and bear weight.
(WMV)

Video S3 **Control mice on treadmill 5 weeks after spinal cord injury.** Mice were assessed for functional recovery, as determined by the use of their left hind limb, at 5 weeks after spinal cord hemisection. Control mice were unable to effectively use their left hindlimb to walk on a treadmill.
(WMV)

Video S4 **Ephrin-A5-Fc-treated mice on treadmill 5 weeks after spinal cord injury.** Mice were assessed for functional recovery, as determined by the use of their left hind limb, at 5 weeks after spinal cord hemisection. Ephrin-A5-Fc treated mice were able to use their left hindlimb to walk on a treadmill, with a step cycle pattern involving use of multiple joints.
(WMV)
