# Supplementary figures and images for: EphA4 Blockers Promote Axonal Regeneration and Functional Recovery Following Spinal Cord Injury in Mice
Source: PLoS One. 2011 Sep 13;6(9):e24636. doi: 10.1371/journal.pone.0024636 (PMC3172248; doi:10.1371/journal.pone.0024636)

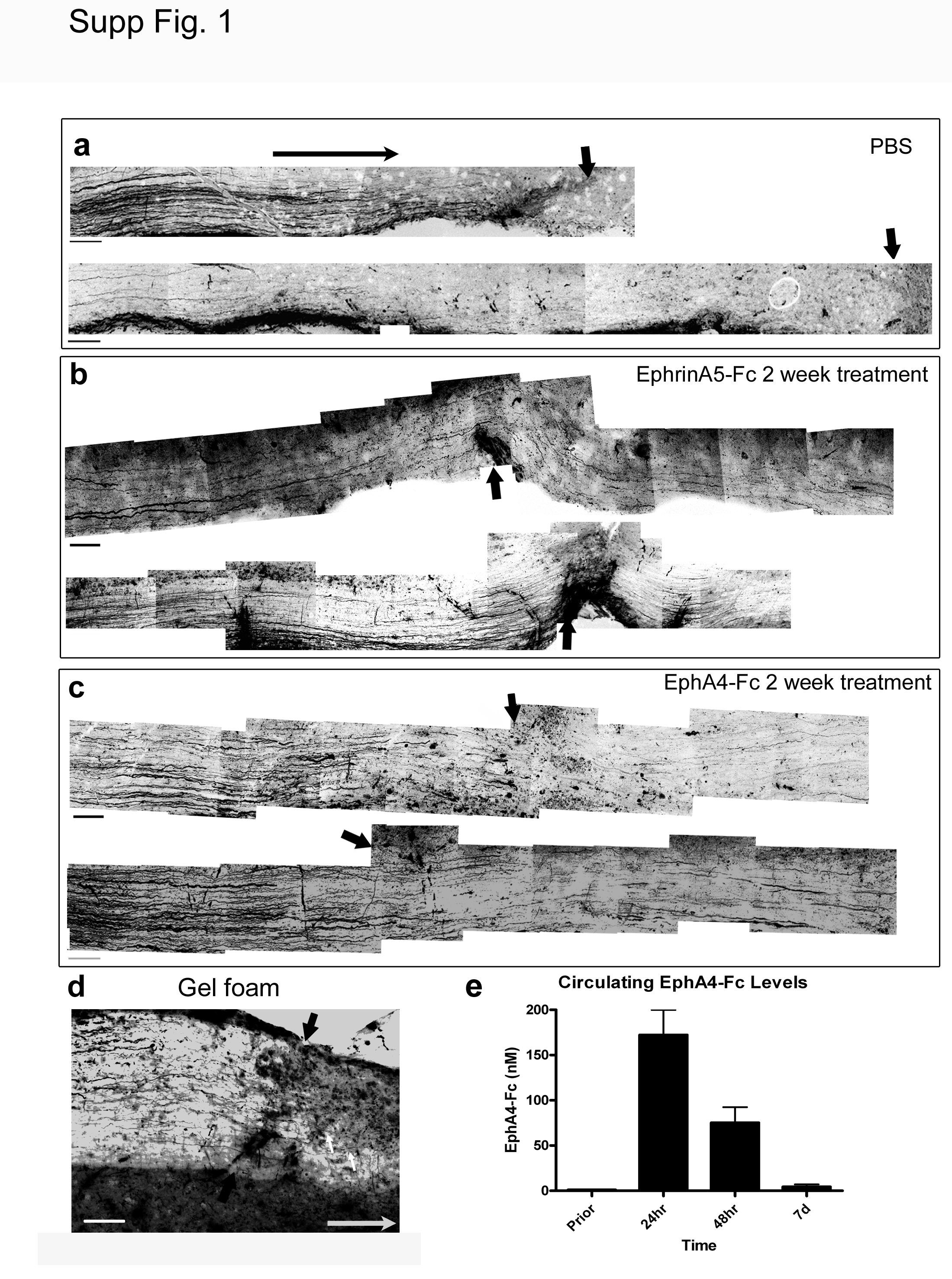

Supplement: Figure S1 — Additional examples of anterograde tracing 6 weeks after spinal cord injury. Spinal cords from (a) PBS control, (b) ephrin-A5-Fc treated and (c) EphA4-Fc treated mice. Arrows indicate lesion site. (d) Local administration of EphA4-Fc does not promote axonal regeneration through the lesion. Immediately following spinal cord injury, 100 µg EphA4-Fc or human IgG was added in a 10 µl solution to 3×4 mm gelfoam and placed directly above the injury site. Local administration of EphA4-Fc by saturated gelfoam resulted in slightly better axonal regrowth than the 1-week i.p. treatment, with many axons entering the lesion site (white arrows), but it was not as effective as the 2-week i.p. treatment. Scale bars in a-c, 50 µm; d, 200 µm. (e) Analysis of EphA4-Fc clearance. EphA4-Fc (1 mg) was injected i.p. and blood samples from three animals were taken at 24 hours, 48 hours and 7 days. One uninjected animal served as a baseline control. Serum was prepared from the blood samples and circulating EphA4-Fc was detected by ELISA. Briefly, purified anti-mouse EphA4 (IF9) monoclonal antibody was bound to EIA plates. Reference EphA4-Fc was diluted from 200 ng/ml to 0 ng/ml and the serum was diluted 1∶1000 and 1∶2000. All standards and samples were added in triplicate. Following washing, bound EphA4-Fc was detected with anti-human IgG-HRP and SIGMAFAST™ OPD colorimetric substrate. A 4th-order polynomial standard curve was generated (r2>0.999) and used to calculate the serum levels of EphA4-Fc. Data are presented as mean±SEM. (TIF) [file pone.0024636.s001.tif]

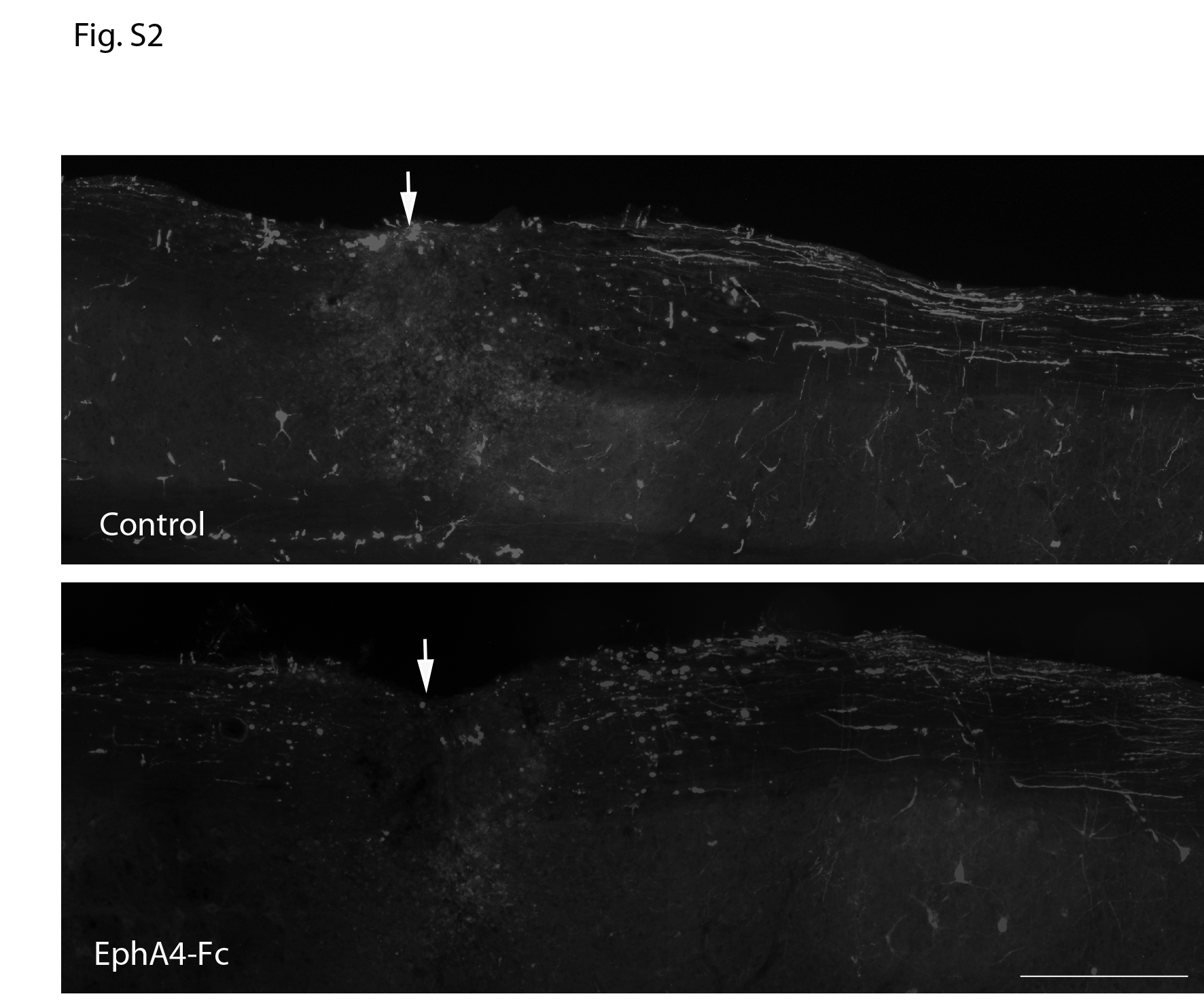

Supplement: Figure S2 — Anterograde tracing of spinal cords that were labeled prior to spinal cord hemisection. Anterograde tracing of spinal cords at 4 days post-injury, labeled 1 week prior to injury shows that there was axonal die-back in control and treated mice. Arrow indicates injury site. Scale bar, 500 µm. (TIF) [file pone.0024636.s002.tif]
